# Autologous mitochondrial microinjection; a strategy to improve the oocyte quality and subsequent reproductive outcome during aging

**DOI:** 10.1186/s13578-019-0360-5

**Published:** 2019-11-29

**Authors:** Halimeh Mobarak, Mohammad Heidarpour, Pei-Shiue Jason Tsai, Aysa Rezabakhsh, Reza Rahbarghazi, Mohammad Nouri, Mahdi Mahdipour

**Affiliations:** 1 Women’s Reproductive Health Research Center, Tabriz University of Medical Sciences, Tabriz, Iran; 2 Department of Clinical Sciences, Faculty of Veterinary Medicine, Ferdowsi University of Mashhad, Mashhad, Iran; 3 Center for Developmental Biology and Regenerative Medicine Research, National Taiwan University/NTU, Taipei, Taiwan; 4 Department of Veterinary Medicine, School of Veterinary Medicine, National Taiwan University/NTU, Taipei, Taiwan; 5 Aging Research Institute, Tabriz University of Medical Sciences, Tabriz, Iran; 6 Stem Cell Research Center, Tabriz University of Medical Sciences, Tabriz, Iran; 7 Department of Applied Cell Sciences, Faculty of Advanced Medical Sciences, Tabriz University of Medical Sciences, Tabriz, Iran; 8 Department of Reproductive Biology, Faculty of Advanced Medical Sciences, Tabriz University of Medical Sciences, Tabriz, Iran

**Keywords:** Oocyte quality, Mitochondrial transfer, Ageing, Reproduction

## Abstract

Along with the decline in oocyte quality, numerous defects such as mitochondrial insufficiency and the increase of mutation and deletion have been reported in oocyte mitochondrial DNA (mtDNA) following aging. Any impairments in oocyte mitochondrial function have negative effects on the reproduction and pregnancy outcome. It has been stated that infertility problems caused by poor quality oocytes in women with in vitro fertilization (IVF) and repeated pregnancy failures are associated with aging and could be overcome by transferring large amounts of healthy mitochondria. Hence, researches on biology, disease, and the therapeutic use of mitochondria continue to introduce some clinical approaches such as autologous mitochondrial transfer techniques. Following mitochondrial transfer, the amount of ATP required for aged-oocyte during fertilization, blastocyst formation, and subsequent embryonic development could be an alternative modality. These modulations improve the pregnancy outcome in women of high reproductive aging as well. In addition to overview the clinical studies using mitochondrial microinjection, this study provides a framework for future approaches to develop effective treatments and preventions of congenital transmission of mitochondrial DNA mutations/diseases to offspring. Mitochondrial transfer from ovarian cells and healthy oocytes could lead to improved fertility outcome in low-quality oocytes. The modulation of mitochondrial bioactivity seems to regulate basal metabolism inside target oocytes and thereby potentiate physiological activity of these cells while overcoming age-related infertility in female germ cells.

## Background

Aging is known to cause complications and time-dependent pathologies in cellular functions which affects most living organisms. The underlying cause of aging is still not fully understood; however, the most prominent theory is a mitochondrial free radical theory of aging (MFRTA) [1–3]. Based on this theory, reactive oxygen species (ROS), and diverse toxic byproducts are produced during aerobic metabolisms which are likely involved in cellular genomes and mitochondrial DNA (mtDNA) injury and mutations. The increased production of ROS causes distinct impairment in the cellular respiratory chain leading to mutation accumulation and imbalanced redox activity [1, 4, 5]. The basal levels of ROS are often declined during the normal functioning phase of the mitochondrial respiratory chain, whereas the surplus amount of cellular ROS is seen by aging, contributing to the significant increase in oxidative damage and the loss of cellular activity [4, 5].

## Aging and mitochondria

One of the most important cellular organelles affected upon aging is mitochondria. The vital function of energy (adenosine 5-triphosphate, ATP) production of mitochondria through oxidative phosphorylation makes it the powerhouse in each cells [6–8]. While mitochondria have been described to take part in the energy metabolism, homeostasis of calcium, growth, and cell apoptosis [9–11], they are also touted as the primary source of intracellular ROS production [6, 12].

Unlike other intracellular organelles, mitochondria have a unique genome known as mtDNA, a double-stranded and circular DNA with approximately 16.5 kb [13]. Due to the lack of genomic repair mechanisms and protective mechanisms of histones structure, the mutation rate of mtDNA is approximately 15 times higher when compared to nuclear genome mutation rate [14, 15]. It has been shown that the elevation of mtDNA somatic mutations per se can be induced by age-related ROS and free radicals accumulation resulting in mitochondrial dysfunction [12, 16]. Mammalian mitochondria are naturally inherited maternally, as the sperm mitochondria are degraded following fertilization in the oocyte in order to protect the embryo and offspring from possible transmission of mutations and heteroplasmy [17, 18]. Although the level of mtDNA heteroplasmic inheritance is low (1–2%) in humans, it has been indicated that this low-level heteroplasmy inheritance along with other inherited mitochondrial and somatic mutations could contribute to the aging-related pathologies [19, 20].

## Relationship between mitochondria and oocyte aging

The major population consulting fertility and assisted reproductive centers are often women of high reproductive age [12]. A decrement in both the quality and the number of oocytes is often associated with advanced maternal reproductive aging and the main reasons for the suboptimal reproductive outcome [12, 21–23]. Either cytoplasmic or nuclear oocyte maturation is essential for successful fertilization and embryonic development [23, 24]. The maturity of oocyte nucleus is characterized by the appearance of the polar body as a result of metaphase II in meiosis division, whereas the evaluation of mitochondrial parameters (such as distribution pattern and activity) is applied to assess the cytoplasmic maturity [24]. Upon aging, the decline in oocyte quality, defects in mitochondrial function as well as the increased level of mutation and deletion in oocyte mtDNA have been reported [25–27]. It has been shown that fertilization potency of the oocytes and subsequent growth potential of the embryos are integral to the number of mtDNA and mtDNA content in elderly women which closely correlate with the production of ATP in developing embryos [12, 21, 28–30]. Due to the higher energy requirements in developing embryos, the reduction of glycolysis and preservation of mtDNA function until blastocyst stage in the oocytes are considered as the major source for supplying the required ATP during the oocyte maturation, cleavage, embryo preimplantation and embryogenesis [6, 17, 31]. It has been demonstrated that defects in mitochondrial biogenesis and ATP production system are associated with meiotic abnormalities, chromosomal aneuploidy, developmental arrest, follicular atresia, and eventually abnormal embryo development [17, 21, 26, 32]. Evidence from animal models showed that increased maternal age declined energy production efficiency in the oocytes which is highly correlated with the accumulation of mitochondrial genome mutations, morphological changes, increasing ROS production and interruption of the respiratory chain [6, 17, 27, 29]. Therefore, the quality and quantity of mitochondria in the oocytes are important and could be essential indicators for successful fertilization and embryo growth [31, 33].

Mitochondria proliferation is initiated in primordial germ cells (PGCs) after migration to ovary niche for the initiation of oogenesis and the number of mitochondria continues to increase during early and later stages of oogenesis process increasing from 200 mtDNA copy number to over 200,000 copies. Therefore, the mature oocyte carries the most mtDNA copies compared to PGCs and immature oocytes [18]. However, owing to the lack of replication after maturation, a decline in intracellular mitochondrial numbers is observed in fertilized oocytes and blastocyst stage of preimplantation embryos. Nevertheless, the mitochondrial replication is resumed after the implantation stage [32]. The microscopic examination of mitochondria distribution and structure in human oocytes during oogenesis and embryogenesis confirmed that the mitochondria in the metaphase I (MI), metaphase II (MII) and fertilized oocytes in cleavage stage have inert appearance with round shape, dense matrix, transverse cristae and are spread throughout ooplasm with close association with smooth endoplasmic reticulum, whereas mitochondria in the morula and blastocysts stages are oval to spherical in shape and tend to be localized around nuclei, indicating the high mitochondrial dynamic, either in number and structure, during embryonic development [34]. The most morphological changes occur at blastocyst stage coincided with mitochondria elongation and development of transverse cristae, indicating that mitochondria are highly active during blastocyst expansion, differentiation and hatching. Significant changes in the mitochondrial distribution also occur in the pronuclear stage post-fertilization [34].

To date, various compounds, including antioxidants and coenzymes (e.g., l-carnitine [35], coenzyme Q10 [36], resveratrol [37], and α-lipoic acid [5, 38, 39]) have been used to improve mitochondrial function and fertility outcome. As aforementioned, the isolation of mitochondria from oocytes of young and healthy donors and transplantation into older and unhealthy oocytes may considerably benefit the rate of ATP production and oocyte quality in general. The mitochondrial transfer has been evaluated by some researchers in mouse, pig, hamster, cattle and human [26, 33, 40–42]. Investigators have suggested that mitochondrial transfer is an alternative treatment approach proposed for transferring healthy mtDNA to the next generation. Following mitochondrial transfer, the basal ATP required for aged-oocyte activation could be achieved during fertilization and blastocyst formation, and thereby contribute to subsequent embryonic development and an improved pregnancy outcome in women of advanced aging as well [12, 29, 43].

## Mitochondrial transfer procedures

Unlike the nuclear genome received from both parents, mtDNA are transmitted exclusively maternally to the embryo in most mammalian species, and any mutations/dysfunctions in maternal mtDNA reservoir could contribute to the most incurable genetic disorders, such as ophthalmoplegia disorders, Kearns-Sayre syndrome, maternally inherited Leigh syndrome, mitochondrial myopathy, Pearson syndrome, Coenzyme Q10 deficiency, mitochondrial DNA depletion syndromes (MDS), benign cytochrome C oxidase deficiency, chronic intestinal pseudo-obstruction (CIPO) etc. in offspring [44–46]. Hence, researchers tend to introduce different therapeutic approaches that involve microscopic manipulation by replacing aberrant mtDNA through mitochondria replacement procedures in the target oocytes to minimize mitochondria-related disorders in the offspring. Some of these techniques are described below.

### Pronuclear transfer (PNT)

**Fig. 1:**
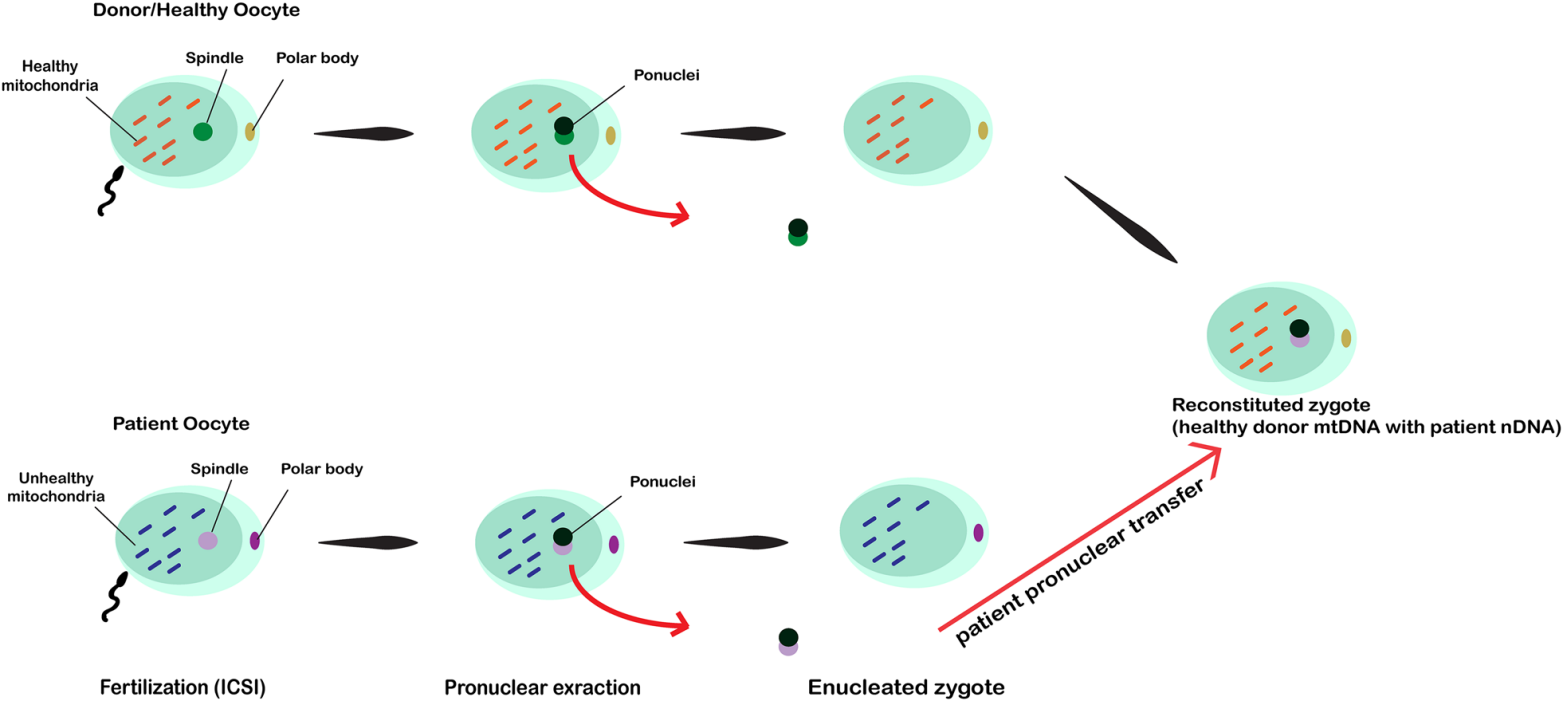
In this technique, first, both recipient and donor oocytes are fertilized using the intended partner’s sperm through ICSI. As soon as the pronuclei is formed, the pronuclei from abnormal mtDNA oocyte is transferred into the enucleated abnormal mtDNA zygote using micromanipulation equipment

During transfer technique (Fig. 1), pronuclei are transferred from one zygote with abnormal mtDNA to another zygote with healthy mtDNA. First, both recipient and donor oocytes should be fertilized by the intended partner’s sperm through intracytoplasmic sperm injection (ICSI) or IVF. Soon after the oocytes are fertilized and pronuclei is formed, the pronuclei from defective oocyte is transferred into the enucleated zygote using micromanipulation equipment. Therefore, the reconstructed zygote will have healthy mtDNA from the donor with original parents nuclear DNA [29, 47]. Results from various studies demonstrated that the use of PNT has a great potential to reduce the transmission of inadequate maternal mtDNA to the offspring. Craven et al. [47] showed that the carry-over of mtDNA after PNT was low (less than 2%) or even non-detectable after cleavage and subsequent development until the blastocyst stage in vitro. This technique, however, is perhaps not fully warranted for the prevention of mtDNA diseases carry-over and should be performed along with prenatal screening [38, 48].

### Spindle transfer

**Fig. 2:**
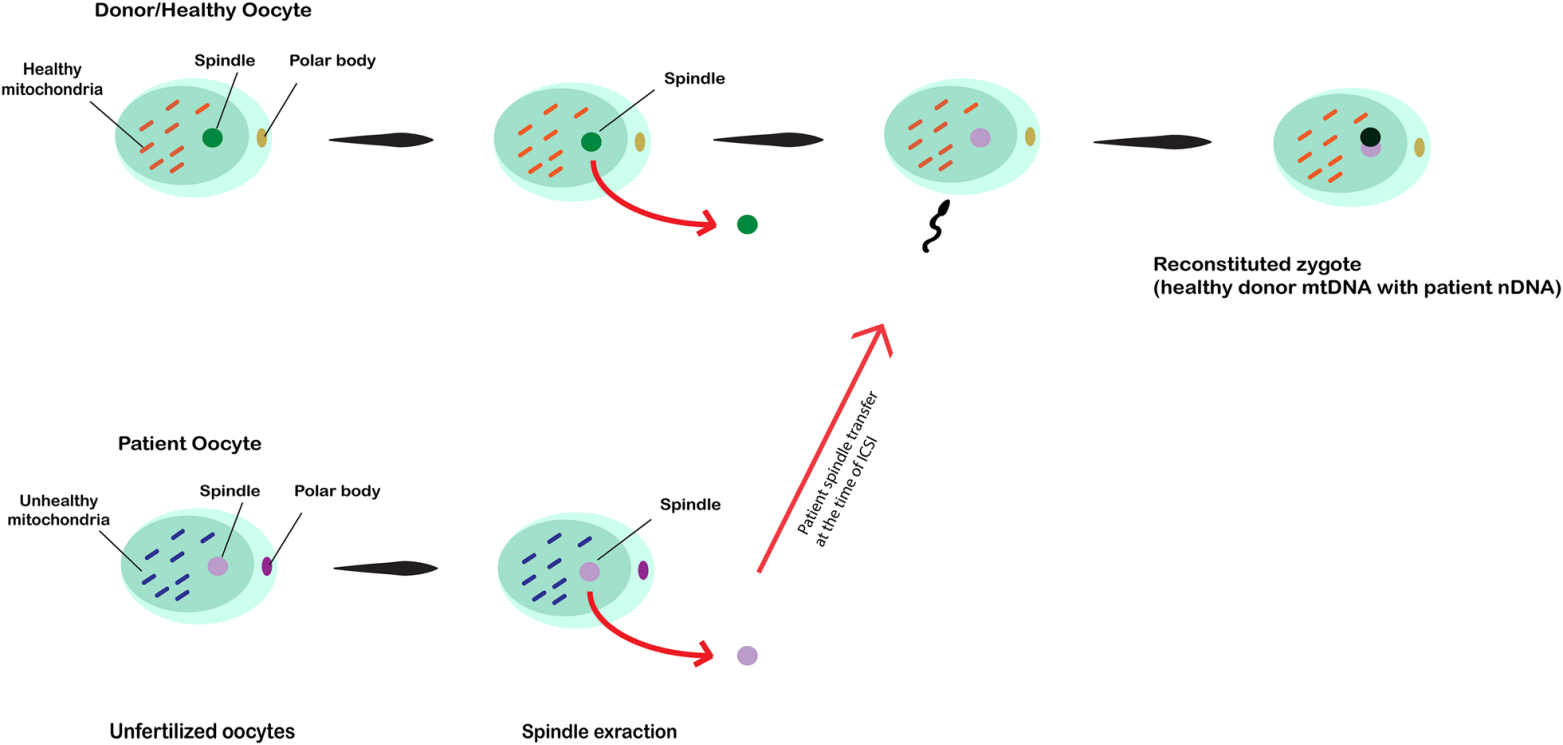
In this technique, the spindle from defective oocytes is extracted and microinjected into donor healthy oocytes in which the spindle is removed. After fertilization by ICSI procedure, the zygote is reconstituted

This technique aims to provide healthy mitochondria from donor oocytes to deficient and/or aged counterparts; the metaphase II spindle was extracted from affected oocytes and transferred into donor healthy oocytes with a discarded spindle. After the fertilization by ICSI, the produced zygote cell contains mitochondria from the healthy donor and nuclear DNA from its original parent [29, 49] (Fig. 2). The first live birth case in human implementing spindle transfer was reported by Zhang et al. [46] in a 36-year-old woman diagnosed with Leigh syndrome with a history of abortion and mtDNA mutation rate of 24.5%, in which a healthy boy in the 37th week of pregnancy was born after spindle transfer. Moreover, a study conducted by Tachibana et al. [50] showed live birth of three healthy offspring in non-human primate using spindle transfer technique by the genetic analysis. They successfully detected both donor oocyte nuclear DNA and original parent oocyte’s mtDNA in the subsequent progeny. By using spindle transfer, it is estimated that mtDNA carryover to be less than 1% and has been introduced to minimize the transfer of congenital malformations of mtDNA to offspring [48, 51].

### Blastomere transfer

**Fig. 3:**
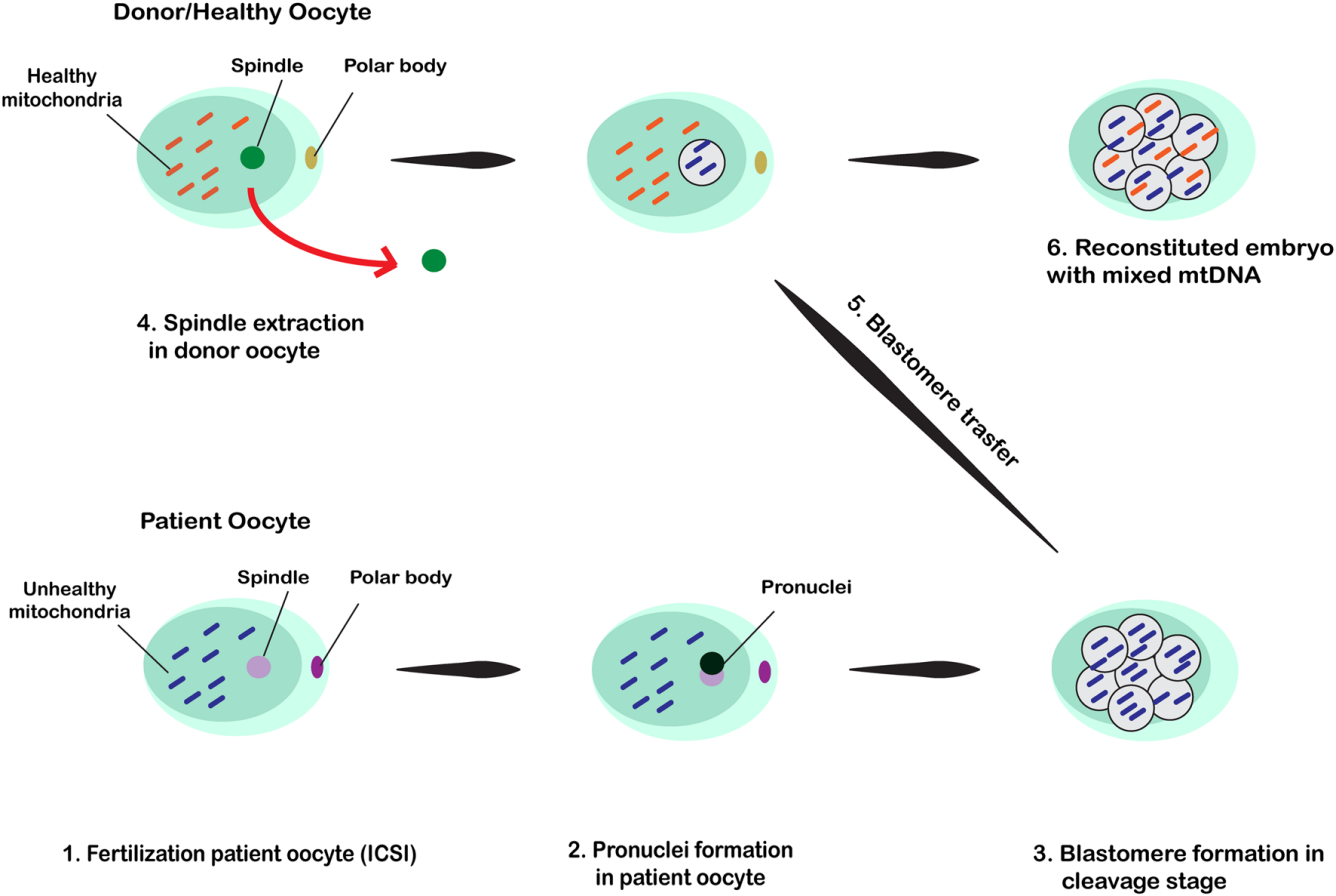
The affected oocytes with abnormal mitochondria are fertilized through either IVF or ICSI and as a result of cleavage, blastomeres are formatted. A blastomere is transplanted into the healthy donor oocyte which its spindle had already been removed. Then, the embryo is reconstituted after blastomere transfer

This technique is performed following the fertilization of affected oocyte containing defective mitochondria through either IVF or ICSI and blastomere formation after cleavage. A blastomere is transplanted into the healthy donor oocyte that its spindle had already discarded. Eventually, the embryo is reconstituted after blastomere transfer (Fig. 3). No clinical studies have been carried out regarding the effectiveness of blastomere transfer in human; however, controversial studies using this method such as heteroplasmic babies are described in animal studies [29, 52].

### Ooplasmic or cytoplasmic transfer

**Fig. 4:**
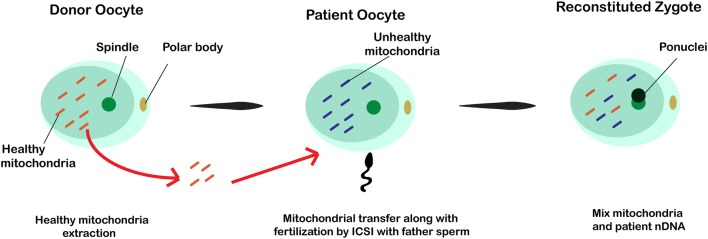
In this procedure a fraction of the donor oocyte’s cytoplasm containing healthy mitochondria is injected into the recipient oocyte’s cytoplasm using ICSI procedure along with sperm. After cytoplasmic transfer, the reconstituted zygote contains parent nuclear DNA and mixed mtDNA from both donor and recipient oocytes

This method involves the simultaneous transfer of a fraction of the donor oocyte’s cytoplasm containing healthy mitochondria along with sperm to the recipient oocyte’s cytoplasm using ICSI procedure. In this case, the reconstituted zygote contains the original parent nuclear DNA and mixed mtDNA from both donor and recipient oocytes (Fig. 4). It is believed that the donated portion of ooplasm possesses healthy mitochondria and other beneficial cytosolic factors for promoting the development of oogenesis and embryogenesis [28, 53, 54]. However, this technique is not considered as a primary clinical technique due to the possibility of the heteroplasmy and chromosomal abnormalities in the subsequent embryos [29, 55].

### Polar body transfer (PBT)

In addition to previously described mitochondrial transfer techniques, polar body (PB) transfer is another method which has been investigated intensively. PB transfer has some advantages, such as minimal transfer of patient mtDNA to the intended embryos due to the lower content of mitochondria in the PB. Moreover, PBs can be easily observed and sampled by using a micro-pipette without damaging the chromosomes [56, 57].

**Fig. 5:**
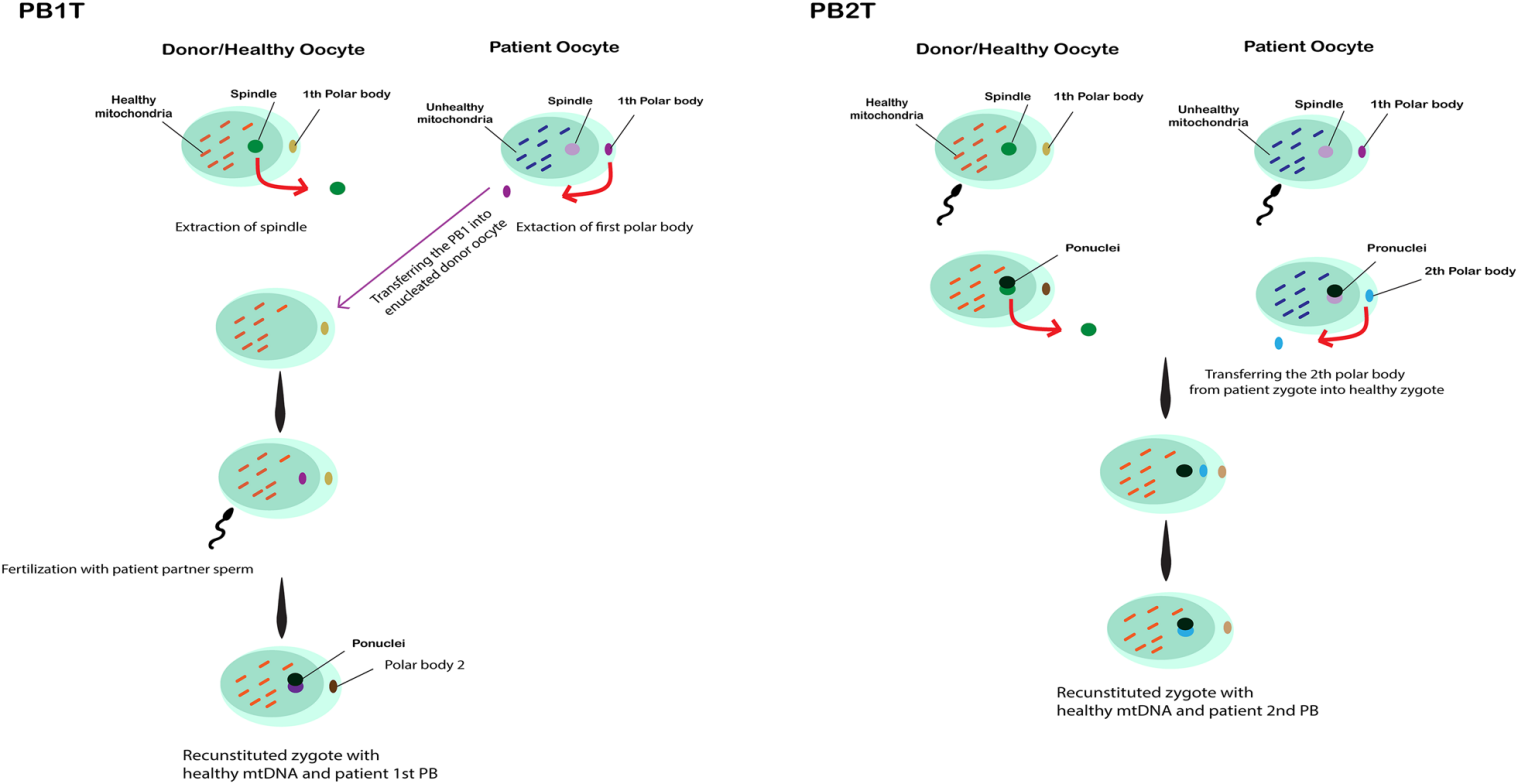
In PB1 transfer procedure, after enucleating the recipient oocyte, PB1 from donor oocyte is discarded, then removed PB1 is fused to enucleated recipient oocyte. After that, the ICSI procedure is performed with partner’s sperm. In PB2 transfer procedure, following both donor and recipient oocytes fertilization, PB2 from donor oocyte is extracted and transferred into the recipient zygote in which the female pronuclei is removed. Eventually, the reconstructed zygote is produced

PB transfer is performed in two subsequent procedures: Polar body 1 (PB1T) and polar body 2 (PB2T) transfer (Fig. 5).

- PB1T: after enucleating the recipient oocyte, PB1 is isolated from donor’s mature MII oocyte. Extracted PB1 is fused to enucleated recipient oocyte. Then, ICSI procedure is performed with the intended partner’s sperm which will result in reconstituted oocyte [57].
- PB2T: following fertilization of both donor and recipient oocytes, second polar body from the original mother is extracted and injected into the recipient zygote in which the female pronuclei is removed. As a result, the reconstructed zygote is generated [57].

### AUGMENT technology

**Fig. 6:**
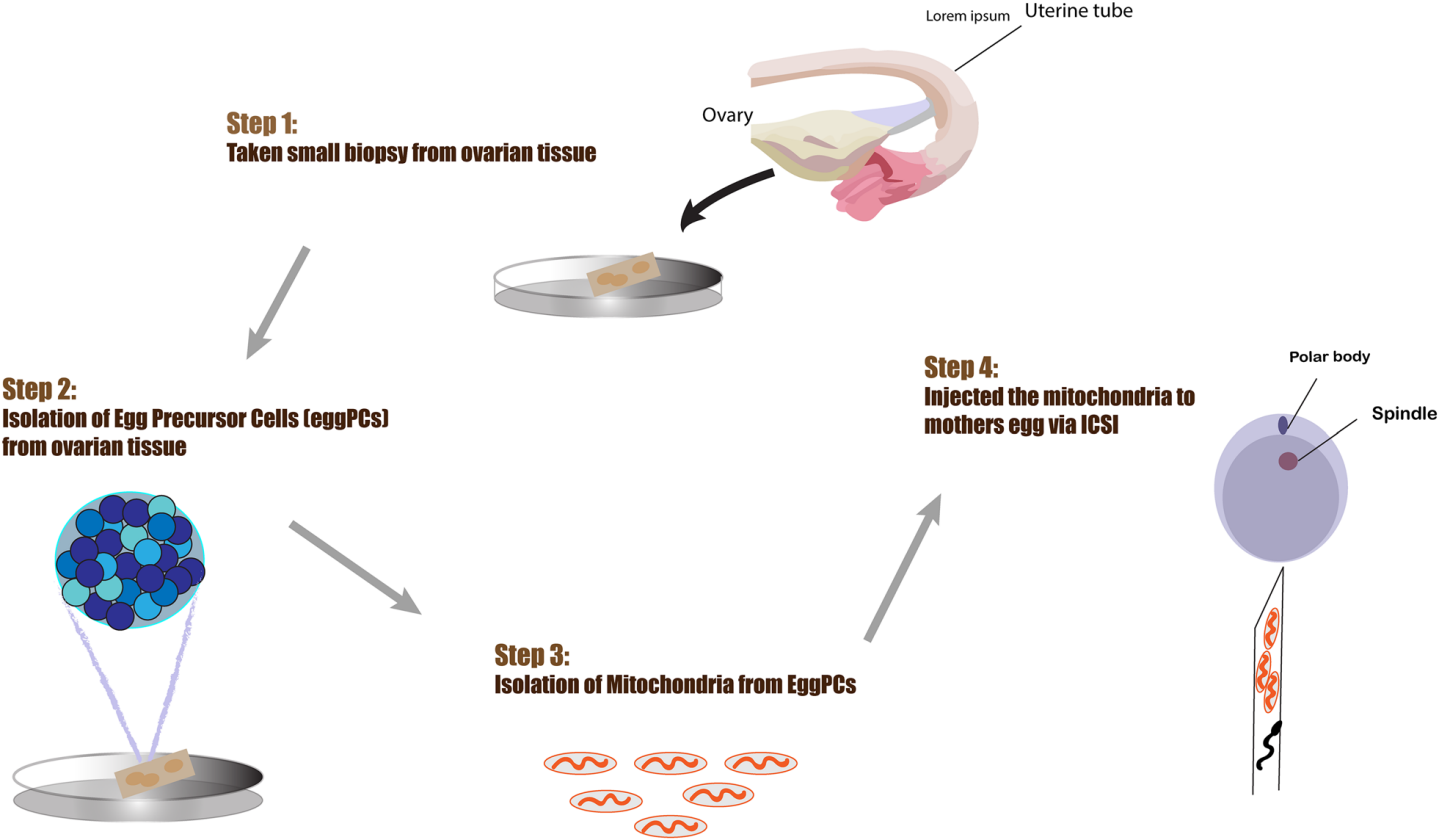
In this procedure first, the biopsy is made from ovarian cortex and egg precursor cells (egg PCs) are isolated from biopsied ovarian tissue followed by mitochondria isolation. Eventually, the isolated autologous mitochondria are microinjected into affected oocytes through ICSI

Autologous germline mitochondrial energy transfer (AUGMENT) is the strategy launched by OvaScience in 2014 to transfer energy-producing mitochondria from autologous egg precursor cells into the eggs (Fig. 6). This method is implemented to enhance oocyte competence and increase the level of energy production required for oocyte maturation, normal embryonic development, and successful fertilization via providing the adequate quality, number, and function of mitochondria [21, 58, 59]. Based on reports of clinical studies in both human and animals, autologous mitochondria is injected cytoplasmically during the ICSI procedure through AUGMENT treatment. This action has been shown to enhance the quality of the egg and fetus [17, 21, 58]. The mitochondrial population is extracted for AUGMENT treatment is known to be highly energetic with a minimum level of deletion and mutations and as these mitochondria are separated from patient’s own cells. As a matter of fact, the probability of heteroplasmy in the offspring and presence of the third genetic material need scientific approval. It is noteworthy to mention that the introduction of exogenous materials and allogenic mitochondrial mass to the target oocytes must be evaluated regarding cellular immunity. Calling attention, some studies however showed that patients with multiple previous IVF failure might not be the good candidates for mitochondrial transplantation [17, 59–61].

## Results of clinical studies following mitochondrial transfer

Most studies in animal and human setups have proved that ATP content and mtDNA copy numbers are relatively lower in aged oocytes when compared with young oocytes [43, 62, 63]. Simsek-Duran et al. [43] reported that the number of mitochondria declines in mouse and hamster oocytes during aging, and as a consequence, the ATP production falls to 35.4% and 38.4%, respectively. Structural changes such as spherical to an elongated shape, vacuolization, cytoplasmic lamellae alterations, and cristae changes were also identified in the mitochondria during aging using transmission electron microscopy (TEM).

Li et al. [41] transplanted the autologous platelet mitochondria from young hamster into aged hamster oocytes with the aim of examining the biochemical and morphological changes of mitochondria in young and old hamster’s oocytes and to increase oocyte quality after mitochondrial microinjection. The results indicated that ATP level and mtDNA copy number decreased by 21% and 56% respectively in elderly hamsters. Morphological changes and declined mitochondrial numbers were simultaneously observed in older oocytes in comparison with young hamster oocytes. An improved fertility rate was seen with increased blastocyst formation after IVF with autologous mitochondrial microinjection in aged oocytes. Li et al. suggested that platelets contain a low level of defective mitochondria due to high turnover rates (approximately 10 days) and could be considered as a good source of healthy mitochondria source compared to autologous granulosa cells in which their mtDNA mutations and deletions have been reported to increase during aging [16, 31, 62, 64]. In this respect, Kristensen et al. [12] have observed that oogonial stem cells (OSCs) are also not a suitable mitochondria source due to their low mitochondrial number as well as practical extraction difficulties.

Considering other cell sources for mitochondrial transfer, Tzeng et al. [65] injected the autologous cumulus granulosa cells (cGCs)-derived mitochondria into poor quality oocytes in patients with failed IVF/ICSI and evaluated the pregnancy outcome. The results revealed that the pregnancy rate in mitochondrial and non-mitochondrial transfer groups was 35.2% and 6.2%, and the abortion rate was 15.4% and 100%, respectively. On day 3, embryo quality and fertilization rate were much better in mitochondrial transfer group compared to non-mitochondrial transfer group. Moreover, the DNA fragmentation and cellular apoptosis rates were decreased and twenty live births, including 7 twins and 2 ectopic pregnancies were confirmed after cGCs-derived mitochondrial transfer procedure. In another trial, Kong et al. [66] observed that co-injection of the autologous GCs-derived mitochondria and sperm during ICSI in oocytes improved embryo quality and pregnancy rate (7 clinical pregnancy out of 18 cases in > 37 years old women). However, fertilization rates were not statistically different between the control and treatment groups.

In line with the concept between aging and mitochondria quality, Fragouli et al. compared the mtDNA quantity in blastocysts and cleavage stage embryos between young and old patients. The results showed the high level of aneuploidy in blastocysts as well as low level of mtDNA content in cleavage stage embryos from older patients [6, 49]. Poor quality oocytes have shown to contain low mtDNA copy number than good quality oocytes [33]. Hua et al. indicated that the morula, blastocyst, hatched blastocysts rates, and blastomere numbers in good quality oocytes (G-oocytes) and poor quality oocytes along with the mitochondria transfer (P-oocytes + MIT) are higher than poor quality oocytes (P-oocytes) without GCs-derived mitochondria transfer in bovine moldel while there is no difference between G-oocytes and P-oocytes + MIT. So, transferring mitochondria isolated from GCs of a similar breed of cattle could improve the quality of embryo during preimplantation development [33, 63]. In another study conducted by Igarashi et al., they observed that mitochondrial membrane potential, oxygen consumption levels and mitochondrial transcriptional factor A (TFAM, a regulator of mitochondrial biogenesis) expression in young oocytes are higher in compared to aged oocytes. It has been reported that above-mentioned defects are associated with a decrease in ATP production, mitochondrial function, oocyte function, embryo development, viability, and ultimately poor pregnancy outcome [67–69]. Intriguingly, in another study by Igarashi et al., they did not observe any improvement in embryo development and fertilization rate in aged oocytes following the mitochondrial transfer. These authors attributed these findings to a possible defect in other important cytoplasmic organelles or factors within the oocyte, such as the endoplasmic reticulum and suggested that these organelles could also play a role in embryonic development [67]. In contrast to human study, Yi et al. injected the mice hepatocytes-derived mitochondria into the 2PN stage zygotes of young and older mice. The results showed that the development of the embryo to blastocyst stage has been remarkably improved in young mice (37.65% versus 20.91% in injected and control groups, respectively). In older mice, 54.35% and 18.92% zygotes developed to morula stage and, 43.48% and 8.11% zygotes developed to the blastocyst stage in injected and control groups, respectively. These findings illustrated that the mitochondrial transfer at the zygote stage from older mice could significantly enhance the development to morula and blastocyst stages unlike human zygots [22, 63].

Since August 2015, two major fertility centers [FAKIH IVF (Dubai, United Arab Emirates) and the Toronto Center for Assisted Reproductive Technologies (TCART; Toronto, Canada)] have set up and developed AUGMENT as a therapeutic practice for women with poor reproductive performance. The data collected based on 93 patients following AUGMENT treatment from both centers showed that AUGMENT significantly increased pregnancy rates up to 3- to 6-fold in TCART and FAKIH IVF centers, respectively. Following 104 cycles of AUGMENT in these two centers, 4 live births from 6 babies were born which is comparable with 5 live births during 369 cycles of IVF without AUGMENT. Taken together, the results of these two centers demonstrated the clinical efficacy of mitochondrial transfer protocol for aged patients to improve human reproductive performance [21, 58]. Recent studies have shown the existence of oogonial precursor cells (OPCs) in the human ovaries as one of the autologous sources of “healthy mitochondria”. With this respect, Oktay et al. investigated the clinical effectiveness of injected OPCs-derived autologous mitochondria via ICSI in order to improve the quality of oocyte in women with multiple IVF failure. Following autologous mitochondrial injection (AMI), high fertilization rates and embryonic scores were observed which clearly reflects the improvement of oocyte quality. In this particular study, 10 women with the mean age of 34.7 were enrolled and subjected to mitochondrial transfer with remarkable improvement of fertilization rates (49.7 pre-AMI vs 78.3 post-AMI) and better embryo grades (2.3 pre-AMI vs 3.1 post-AMI) resulted in four clinical pregnancies was observed [25].

Barritt et al. [70] traced the transferred donor mitochondria following ooplasmic transfer in the blood samples of human babies by mtDNA fingerprinting and confocal microscopy after donor ooplasm staining. The results showed the distribution and replication of injected donor mitochondria after 24 and 48 h by the ooplasmic transplantation in preimplantation embryos. Also, the authors observed heteroplasmy in baby’s blood. This paper was the first report in human germline genome modification resulting in healthy babies. Taken together, active and healthy mitochondria from healthy fertile donor oocytes were transplanted into the recipient patient oocytes during the ooplasmic transplantation technique resulted in approximately 30 live births worldwide.

Dale et al. [54] described the successful employment of oocyte cytoplasmic transfer to patients who suffered from idiopathic infertility due to a high level of embryonic fragmentation after IVF or ICSI. In this report, a fraction of oocyte cytoplasm of a 25-year-old woman was injected into oocytes of the patient during ICSI procedure after confirmation of normal metaphase-II maturation and morphology contributed a live birth of healthy twins. This result illustrates the successful application of cytoplasmic transfer for enhancing the oocyte and embryo quality. In a similar study conducted by Huang et al. [71], cytoplasm from donor’s zygote and recipient husbands’ sperms were injected simultaneously into the recipient’s MII oocytes suffering from repeated implantation failure (RIF). Following injection MII oocytes developed to cleavage stage embryos and subsequently transferred to the patient’s uterus. As a pregnancy outcome, five healthy babies from four normal pregnant recipients were born. This results confirmed the enhancement effect of zygote’s cytoplasmic injection into oocytes upon ICSI, in patients with RIF. In an animal study, Chiaratti et al. demonstrated that ooplasmic transfer could rescue the exposed bovine oocytes to ethidium bromide (the mtDNA transcription and replication inhibitor), and promoted embryonic development and heteroplasmic calves birth [28, 72].

In another investigation, a 39-years-old woman with low ovarian reserve and ooplasmic deficiency (6.5 years of treatment history) were received ooplasm of a 27-year-old woman during ICSI with successful fertilization and eventually a healthy baby girl was born in weight of 4356 g [53].

Lanzendorf et al. [73] reported a twin pregnancy in a 35 years old patient with a poor quality embryo history following a cytoplasmic transfer from cryopreserved donor oocytes into patient oocytes, however using this procedure in patients with advanced maternal aging (> 40 years of age) resulted in no positive pregnancies.

In an experiment conducted in animal setup, Wang et al. [8] microinjected the autologous mitochondria isolated from adipose-derived stem cells (ADSCs) from an aged mouse into its own GV oocytes. Examining the mtDNA copy numbers, spindle organization status and chromosomal alignment in MII oocytes in microinjection group revealed an enhancement in the maturation process and decline in aneuploidy rates compared to control group. They also concluded that autologous ADSCs-derived mitochondria microinjection via by ICSI procedure could improve the blastocyst rate (up to 30% vs. 15%), embryogenesis and fertility outcome in aged mice.

In contrary to above-mentioned results, Li et al. [74] demonstrated that heterogeneous ooplasmic transfer to rabbit mature oocytes led to an apparent decline in development rate of blastocysts. However, the homogenous ooplasmic transfer did not impact on fertilization rate and embryonic development. They pointed out that the transmission of ooplasm into defective and low-quality oocytes could improve the fertilization rate and embryonic development, not oocytes with normal quality. The result of mitochondrial and/or ooplasmic transfer studies in the reproductive system is summarized in Table 1.

**Table 1:** Summary of mitochondrial and/or ooplasmic transfer studies in the reproductive system

Species	Ooplasm or mitochondria transfer	Increased fertilization rate	Embryo development	Improved blastocysts rate	Clinical pregnancy	References
Human	Ooplasm	√	√	–	One baby	[53]
	Ooplasm	√	√	√	5 baby	[71]
	Ooplasm	√	√	–	Twin	[73]
	Oocyte mitochondria	√	√	√	–	[70]
	Ooplasm	√	√	–	Twin	[54]
	cGCs-mitochondria	√	√	√	7 twins 2 ectopic pregnancies	[65]
Mouse	Ooplasm	√	√	√	–	[79]
	Mitochondria	√	√	√	–	[80]
	Mitochondria	√	√	√	–	[26]
	Somatic cyto/mito	–	×	√	–	[81]
	Hepatocytes mitochondria	√	√	√	–	[22]
	ADSC-mitochondria	√	√	√	–	[8]
	Ooplasm	√	√	√	√	[11]
	Mitochondria	√	√	√	–	[82]
Cow	Granulosa cells mitochondria	√	√	√	–	[33]
	Ooplasm	√	√	√	–	[28]
Pig	Mitochondria	√	√	√	–	[30]
	Mitochondria	√	√	√	–	[40]
Hamster	Platelet mitochondria	√	√	√	–	[41]
Rabbit	Ooplasm	×	×	×	×	[74]

## Ongoing clinical trials around the world about the AUGMENT technique

To the best of our knowledge, trials and clinical application of mitochondrial transfer increasingly being performed for fertility and reproduction competence. In a study compiled by Ishii et al. [75], different authorities have been studied mitochondrial manipulation techniques in clinical setting. In this respect, different institutes from sixteen countries were selected to analyze the legal status of egg donation and genetic modification and discussed implications associated with implementation of current experimental approaches in reproductive medicine. The current clinical trials conducted in relation to AUGMENT treatment in women (ClinicalTrials.gov^1^) are summarized in Table 2. The principal objective of these studies is to increase oocyte and embryo quality in patients or elder women with the history of IVF failure autologous microinjection of mitochondria from different sources. Data showed that mitochondrial source are either ovarian stem cells, GCs, and bone marrow mesenchymal stem cells in these clinical studies. The central consensus in these experimentations are the deterioration of oocyte mitochondrial function and reduced mitochondrial copy number as a major consequence of maternal aging which has significant negative effects on oocyte maturation and embryogenesis. Hence, the primary goal of these trials is to enrich the energy sources in affected oocytes via AUGMENT treatment.

**Table 2:** In progress clinical trials about AUGMENT treatment around the world

Title and ClinicalTrials.gov identifier	Interventions and conditions	Primary outcome measures	Secondary outcome measures	Inclusion criteria	Sponsor	The last update posted	Phase	Status
Clinical application of autologous mitochondria transplantation for improving oocyte quality NTC number: NCT03639506	Procedure: autologous mitochondria transplantation combination Product: autologous mitochondria from BMSCs into oocyte as well as ICSI Condition(s): repetition failure	The rate of live birth	Retrieved oocytes number Fertility rate Clinical pregnancy rate Good quality embryo rate normal fertility rate	Inclusion criteria Women age 20–43 years AMH (level ≥ 1.1 ng/mL) BMI: 18–25 kg/m^2^ Previous failed transfer cycles ≥ 2 Written informed consent Exclusion criteria Abnormal uterine development, endometrial adhesion or previous endometrial dysplasia Other medical diseases that cannot be pregnant Complicated with malignant tumors or precancerous disease, endometriosis Untreated hydrosalpinx The man has definite factors that affect the quality of the embryo, such as non-obstructive spermatozoa and persistent abnormal DNA fragment rate (> 30%) ICSI with donor Eliminate or falls off criteria The number of retrieved oocytes < 6	Sun Yat-sen University	August 2018	–	Not yet recruiting
Autologous mitochondrial transfer in ICSI to improve oocyte and embryo quality in IVF patients. Pilot study NTC number: NCT02586298	Autologous mitochondria with ICSI Condition(s): infertility	Ongoing pregnancy rate	–	Inclusion criteria BMI < 30 Age ≤ 42 years Previous IVF cycle with 5 or more metaphase II oocytes after retrieval Serum AMH ≥ 4 pM/L Will undergo an IVF cycle with arrays in preimplantation genetic screening Semen sample with concentrations exceeding 3 million/mL progressive motile sperm All cases without embryo transfer due to the presence of a blockage of embryo development before D3 in embryos All cases without embryo transfer due to any chromosomal abnormality detected by PGD or PGS techniques A history of previous cycles of IVF with embryo transfer and without pregnancy due to low embryo quality Exclusion criteria Severe male factor Formal contraindication in ovarian cortex biopsy or follicle puncture Any characteristic incompatible with carrying out a new IVF cycle at IVI Valencia	IVI VALENCIA, Spain	August 2017	–	Completed
Improving the reproductive outcome of poor quality ova by injection of autologous somatic mitochondria NTC number: NCT01631578	Injection of autologous mitochondria into the oocytes Condition(s): low ovarian reserve and poor quality oocytes	Oocyte fertilization rate The quality of early embryonic development	Clinical pregnancy	Inclusion criteria Adequate sperm source Adequate uterine cavity And one or more of the following: 3 unsuccessful IVF cycles or > 75% of embryos with < 50% fertilization rate or > 20–50% fragments in previous cycles Age > 40 Exclusion criteria Inadequate sperm source Inadequate uterine cavity	Hadassah medical organization	August 2015	1 2	Withdrawn

## Ethical issues

Numerous in vitro studies have been carried out on transferring the mitochondria between cells to promote the restoring process in affected target cells through intracellulary and extracellulary mechanisms in the various fields including reproduction. Because of some ethical concerns related to mitochondrial transfer procedures, such as the birth of a baby from a third parent, transmitting of the genetic disorders and the donation of mitochondria from other individuals, the mitochondrial transplantation has been considered controversial. Despite these limitations, the AUGMENT mitochondrial transfer has been introduced as an appropriate method for transferring healthy autologous mitochondria to circumvent the ethical issues and legal concerns. On the other hand, more randomized controlled clinical trials are demanded to further examine and validate the ethical and safety concerns [59, 61, 76].

## Conclusion and future directions

The association between ROS, aging, and metabolic abnormalities have been clearly illustrated 60 years ago [4]. In addition to factors related to aging, other factors correlated with poor quality oocytes such as overweight, polycystic ovary syndrome (PCOS), type II diabetes, and other environmental and genetical deficiencies have also been indicated. Oocytes of patients suffering from above-mentioned disorders contain a large number of mitochondrial malformations including structural and functional changes [17, 58, 77]. Due to the strong association between mitochondria and oocyte quality, subsequent embryonic development and fertility are likely to be affected directly by the quality of the mitochondria and mtDNA. Therefore, impairment in mitochondrial function and oocyte mitochondrial deficiency may have a negative effect on reproduction and fertility, hence increasing mitochondrial numbers and/or improving mitochondrial function through mitochondrial transfer from high-quality oocyte to low-quality oocyte could lead to improved fertility outcome and benefit female patients suffering from diabetes, recurrent IVF failure and repeated pregnancy failure associated with aging.

It has been proposed that the injection a small fraction of donor’s oocyte cytoplasm containing healthy mitochondria may restore the normal function of oocytes [71]. In the past, the transfer of donor oocyte cytoplasm resulted in some live births; however, considering heteroplasmic concerns nowadays, this technique is less recommended. To further confirm the safety of the procedure, optimal protocols, including maintenance of homoplasmy in the offspring through using of approved sources of mitochondria (donor mitochondria) without deletions or mutations in mtDNA, or implementation of ovarian origin egg-precursor cells should be established and considered [25, 32, 78]. OvaScience has reported that egg precursor cells (EPCs)-derived mitochondria from germline and unipotent cells in the outer ovarian cortex are the most potential and high-quality sources of healthy and active mitochondria to be used in AUGMENT technique. Therefore, the evaluation of healthy and active mitochondria prior to oocyte reconstruction procedures can be helpful for the selection of fertilizable oocytes [30]. As mitochondria are vital for oocyte maturation and embryo development, mitochondria assessment and application of appropriate technique for mitochondria injection into the defective oocyte are urgently needed especially in women at the risk of disorders associated with aging.

## Data Availability

Not applicable.

## Abbreviations

MFRTA: mitochondrial free radical theory of aging
ROS: reactive oxygen species
MtDNA: mitochondrial DNA
PGCs: primordial germ cells
CIPO: chronic intestinal pseudo-obstruction
MDS: mitochondrial DNA depletion syndromes
PNT: pronuclear transfer
ICSI: intracytoplasmic sperm injection
IVF: in vitro fertilization
PBT: polar body transfer
AUGMENT: autologous germline mitochondrial energy transfer
OSCs: oogonial stem cells
CGCs: cumulus granulosa cells
AMI: autologous mitochondrial injection
ADSC: adipose tissue-derived stem cells
EPCs: egg precursor cells

## Footnotes

1 ClinicalTrials.gov known as a database of general and private clinical studies that have been conducted around the world and a clinical studies source founded by the United States NLM (National Library of Medicine).
